# (*Z*)-5-(3,4,5-Tri­meth­oxy­styr­yl)-2,3-di­hydro­thieno[3,4-*b*][1,4]dioxine

**DOI:** 10.1107/S1600536814004437

**Published:** 2014-03-05

**Authors:** Yu-Tao Liu, Gang Chu

**Affiliations:** a Department of Pharmacy, Yantaishan Hospital, Yantai 264000, Shandong, People’s Republic of China; b Nanjing Sanhome Pharmaceutical Co. Ltd, Nanjing 210038, Jiangsu, People’s Republic of China

## Abstract

In the title compound, C_17_H_18_O_5_S, an analogue of the potent anti­cancer agent combretastatin A-4, the alkene C=C bond has a *cis* conformation and the C—C=C—C torsion angle is 9.0 (3)°. The dihedral angle between the benzene and thio­phene rings is 54.07 (4)°. The dioxene ring adopts a half-chair conformation, with the C atoms of the methyl­ene groups displaced by −0.325 (2) and 0.341 (3) Å from the plane of the other atoms. The C atoms of the two *meta*-meth­oxy groups are close to being coplanar with their attached benzene ring [displacements = −0.025 (2) and −0.196 (2) Å], whereas the C atom of the *para*-meth­oxy group is significantly displaced [by −1.107 (2) Å]. In the crystal, C—H⋯O hydrogen bonds link the mol­ecules into [0-11] chains, which feature two different types of *R*_2_^2^(6) loops.

## Related literature   

For background to combretastatin [systematic name: (*Z*)-2-meth­oxy-5-(3,4,5-tri­meth­oxy­styr­yl)phenol], see Pettit *et al.* (1987 ▶, 1995 ▶); Dark *et al.* (1997 ▶); Thorpe *et al.* (2003 ▶); Tozer *et al.* (2005 ▶). For further synthesis details, see Mohannkrishnan *et al.* (1999 ▶).
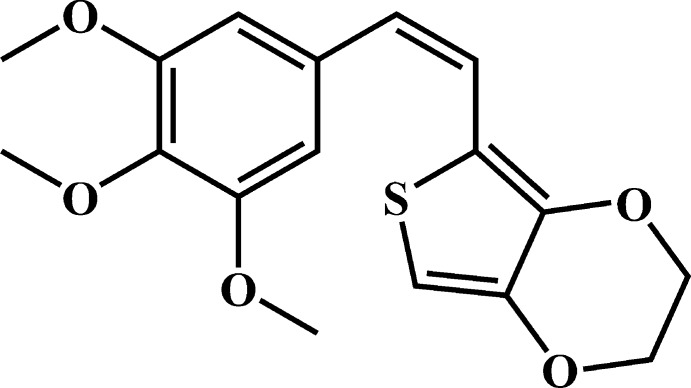


## Experimental   

### 

#### Crystal data  

C_17_H_18_O_5_S
*M* *_r_* = 334.37
Triclinic, 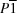

*a* = 8.197 (2) Å
*b* = 8.4527 (15) Å
*c* = 11.835 (3) Å
α = 88.774 (1)°
β = 85.484 (3)°
γ = 76.422 (2)°
*V* = 794.6 (3) Å^3^
*Z* = 2
Mo *K*α radiation
μ = 0.23 mm^−1^
*T* = 296 K
0.30 × 0.28 × 0.26 mm

#### Data collection  

Bruker SMART CCD diffractometer
Absorption correction: multi-scan (*SADABS*; Bruker, 2008 ▶) *T*_min_ = 0.963, *T*_max_ = 0.985
4342 measured reflections
3059 independent reflections
2766 reflections with *I* > 2σ(*I*)
*R*_int_ = 0.159

#### Refinement  

*R*[*F* ^2^ > 2σ(*F* ^2^)] = 0.066
*wR*(*F* ^2^) = 0.179
*S* = 0.99
3059 reflections
212 parameters
H-atom parameters constrained
Δρ_max_ = 0.57 e Å^−3^
Δρ_min_ = −0.69 e Å^−3^

### 

Data collection: *SMART* (Bruker, 2008 ▶); cell refinement: *SAINT* (Bruker, 2008 ▶); data reduction: *SAINT*; program(s) used to solve structure: *SHELXS97* (Sheldrick, 2008 ▶); program(s) used to refine structure: *SHELXL97* (Sheldrick, 2008 ▶); molecular graphics: *SHELXTL* (Sheldrick, 2008 ▶); software used to prepare material for publication: *SHELXL97*.

## Supplementary Material

Crystal structure: contains datablock(s) I, New_Global_Publ_Block. DOI: 10.1107/S1600536814004437/hb7200sup1.cif

Structure factors: contains datablock(s) I. DOI: 10.1107/S1600536814004437/hb7200Isup2.hkl

Supporting information file. DOI: 10.1107/S1600536814004437/hb7200Isup3.cdx

Supporting information file. DOI: 10.1107/S1600536814004437/hb7200Isup4.cml

CCDC reference: 988810

Additional supporting information:  crystallographic information; 3D view; checkCIF report

## Figures and Tables

**Table 1:** Hydrogen-bond geometry (Å, °)

*D*—H⋯*A*	*D*—H	H⋯*A*	*D*⋯*A*	*D*—H⋯*A*
C15—H15*B*⋯O1^i^	0.96	2.57	3.477 (3)	157
C17—H17*B*⋯O3^ii^	0.96	2.57	3.266 (3)	129

Symmetry codes: (i) 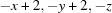; (ii) 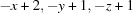.

**Fig. 1.**
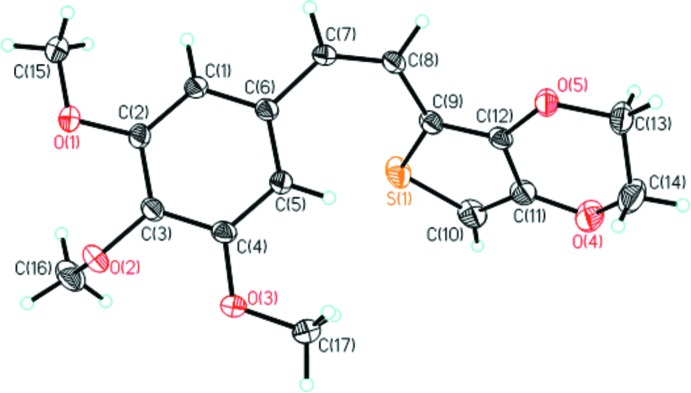
View of the title compound, showing 50% probability ellipsoids.

**Fig. 2.**
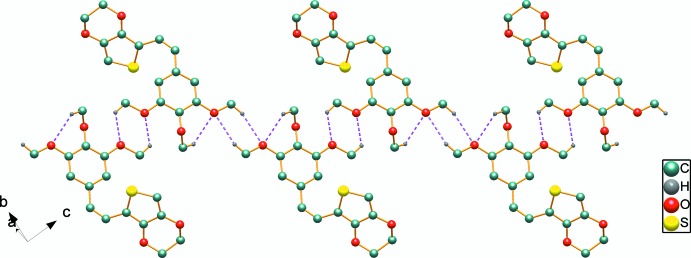
A hydrogen-bonded chain in the title compound.

## supplementary crystallographic information

### 1. Comment

Combretastatins are natural products with extraordinary anticancer activity, which were isolated from tree *Combretum caffrum* (Pettit *et al.*1987). Among these compounds, combretastatin A-4 ((*Z*)-2-methoxy-5-(3,4,5-trimethoxystyryl)phenol, CA-4) possesses the most potent antitumor activity as a result of specifically targeting the vasculature of tumors. (Pettit *et al.* 1995; Dark *et al.* 1997). Its disodium phosphate prodrug has already developed and entered clinical trials (Thorpe *et al.*2003; Tozer *et al.* 2005). Therefore, CA-4 is a very attractive leading compound because of the relatively simple structure and the strong potency against a broad spectrum of human cancer cell lines. The title compound is a new CA-4 analogue, which was prepared by the Wittig reaction (Mohannkrishnan *et al.* 1999). The antitumor activities of the title compound against HL-60, SMMC-7721 and A549 cancer cell lines in vitro are closely to the natural CA-4. We now report the structure of the title compound (Fig. 1). The C=C bond length is 1.335 (2) Å and two aryl units are on the same sides of the double bond plane. Therefore, it exists as *Z* configuration. The planes of the two aryl units, benzene ring and thiophene ring, in the molecule make a dihedral angle of 54.07 (4)°. Therefore, the structure of the title compound is very similar as that of the natural combretastatin A-4 (CA-4). In the crystal structure of the title compound, the C—H···O interactions extend the molecules into a zigzag supramolecular array with dangling thiophene rings (Fig. 2).

### 2. Experimental

To the bromotriphenyl(3,4,5-trimethoxybenzyl)phosphonium salt (5.2 g,10.0 mmol) in dry THF (60 ml) at -78 °C, was added 2.5 Mn-BuLi in hexane (4.0 ml,10.0 mmol) with stirring. After 20 min., a solution of 2,3-dihydrothieno[3,4-*b*][1,4]dioxine-5-carbaldehyde (1.7 g,10.0 mmol) in dry THF (20 ml) was added and resulting mixture stirred at room temperature for 2 h. Then, the mixture was poured into crushed ice, extracted with ethyl acetate (3*30 ml), dried with MgSO_4_, and concentrated *in vacuo*. The resulting solid was purified by column chromatography (petroleum ether/ ethyl acetate, V:V = 5:1) to give white product (1.38 g). Yield: 41.9%. mp. 122–123 °C. The ^1^H NMR, ^13^CNMR, MS spectra and elemental analysis are in accord with the assigned structures. Colourless blocks of the title compound were obtained by the slow evaporation of a petroleum ether/ ethyl acetate solution.

### 3. Refinement

(type here to add refinement details)

### Crystal data

**Table:**

C_17_H_18_O_5_S	*Z* = 2
*M**_r_* = 334.37	*F*(000) = 352
Triclinic, *P*1	*D*_x_ = 1.397 Mg m^−^^3^
Hall symbol: -P 1	Mo *K*α radiation, λ = 0.71073 Å
*a* = 8.197 (2) Å	Cell parameters from 3471 reflections
*b* = 8.4527 (15) Å	θ = 2.5–28.3°
*c* = 11.835 (3) Å	µ = 0.23 mm^−^^1^
α = 88.774 (1)°	*T* = 296 K
β = 85.484 (3)°	Block, colorless
γ = 76.422 (2)°	0.30 × 0.28 × 0.26 mm
*V* = 794.6 (3) Å^3^	

### Data collection

**Table:**

Bruker SMART CCD diffractometer	3059 independent reflections
Radiation source: fine-focus sealed tube	2766 reflections with *I* > 2σ(*I*)
Graphite monochromator	*R*_int_ = 0.159
ω scans	θ_max_ = 26.0°, θ_min_ = 2.5°
Absorption correction: multi-scan (*SADABS*; Bruker, 2008)	*h* = −10→5
*T*_min_ = 0.963, *T*_max_ = 0.985	*k* = −10→10
4342 measured reflections	*l* = −14→14

### Refinement

**Table:**

Refinement on *F*^2^	Secondary atom site location: difference Fourier map
Least-squares matrix: full	Hydrogen site location: inferred from neighbouring sites
*R*[*F*^2^ > 2σ(*F*^2^)] = 0.066	H-atom parameters constrained
*wR*(*F*^2^) = 0.179	*w* = 1/[σ^2^(*F*_o_^2^) + (0.1373*P*)^2^ + 0.0901*P*] where *P* = (*F*_o_^2^ + 2*F*_c_^2^)/3
*S* = 0.99	(Δ/σ)_max_ < 0.001
3059 reflections	Δρ_max_ = 0.57 e Å^−^^3^
212 parameters	Δρ_min_ = −0.69 e Å^−^^3^
0 restraints	Extinction correction: *SHELXL97* (Sheldrick, 2008), Fc^*^=kFc[1+0.001xFc^2^λ^3^/sin(2θ)]^-1/4^
Primary atom site location: structure-invariant direct methods	Extinction coefficient: 0.75 (5)

### Special details

**Table:**

Geometry. All e.s.d.'s (except the e.s.d. in the dihedral angle between two l.s. planes) are estimated using the full covariance matrix. The cell e.s.d.'s are taken into account individually in the estimation of e.s.d.'s in distances, angles and torsion angles; correlations between e.s.d.'s in cell parameters are only used when they are defined by crystal symmetry. An approximate (isotropic) treatment of cell e.s.d.'s is used for estimating e.s.d.'s involving l.s. planes.
Refinement. Refinement of *F*^2^ against ALL reflections. The weighted *R*-factor *wR* and goodness of fit *S* are based on *F*^2^, conventional *R*-factors *R* are based on *F*, with *F* set to zero for negative *F*^2^. The threshold expression of *F*^2^ > σ(*F*^2^) is used only for calculating *R*-factors(gt) *etc*. and is not relevant to the choice of reflections for refinement. *R*-factors based on *F*^2^ are statistically about twice as large as those based on *F*, and *R*- factors based on ALL data will be even larger.

### Fractional atomic coordinates and isotropic or equivalent isotropic displacement parameters (Å^2^)

**Table:**

	*x*	*y*	*z*	*U*_iso_*/*U*_eq_	
C1	0.7976 (2)	0.5843 (2)	0.05341 (13)	0.0332 (4)	
H1	0.7750	0.6054	−0.0219	0.040*	
C2	0.8766 (2)	0.6846 (2)	0.10955 (14)	0.0341 (4)	
C3	0.9126 (2)	0.6527 (2)	0.22295 (14)	0.0346 (4)	
C4	0.8656 (2)	0.5213 (2)	0.27815 (13)	0.0339 (4)	
C5	0.7875 (2)	0.4201 (2)	0.22159 (13)	0.0339 (4)	
H5	0.7591	0.3311	0.2588	0.041*	
C6	0.75215 (19)	0.4526 (2)	0.10883 (13)	0.0319 (4)	
C7	0.6789 (2)	0.3434 (2)	0.04398 (13)	0.0358 (4)	
H7	0.7074	0.3418	−0.0337	0.043*	
C8	0.5779 (2)	0.2464 (2)	0.07919 (14)	0.0362 (4)	
H8	0.5606	0.1761	0.0243	0.043*	
C9	0.4900 (2)	0.2326 (2)	0.19020 (14)	0.0354 (4)	
C10	0.3068 (3)	0.2738 (3)	0.37088 (18)	0.0544 (6)	
H10	0.2395	0.3115	0.4362	0.065*	
C11	0.3539 (2)	0.1146 (3)	0.33906 (15)	0.0439 (5)	
C12	0.4559 (2)	0.0932 (2)	0.23456 (13)	0.0340 (4)	
C13	0.4314 (3)	−0.1752 (2)	0.23211 (18)	0.0548 (6)	
H13A	0.4955	−0.2826	0.2088	0.066*	
H13B	0.3206	−0.1585	0.2040	0.066*	
C14	0.4144 (4)	−0.1663 (3)	0.3584 (2)	0.0671 (7)	
H14A	0.3653	−0.2536	0.3888	0.081*	
H14B	0.5250	−0.1809	0.3865	0.081*	
C15	0.8706 (3)	0.8662 (2)	−0.04727 (16)	0.0465 (5)	
H15A	0.9172	0.7818	−0.1016	0.070*	
H15B	0.9068	0.9631	−0.0697	0.070*	
H15C	0.7500	0.8883	−0.0437	0.070*	
C16	0.9071 (3)	0.9017 (3)	0.3128 (2)	0.0597 (6)	
H16A	0.8508	0.9574	0.2502	0.090*	
H16B	0.9818	0.9629	0.3387	0.090*	
H16C	0.8253	0.8909	0.3734	0.090*	
C17	0.8570 (3)	0.3659 (3)	0.44741 (16)	0.0570 (6)	
H17A	0.7378	0.3771	0.4470	0.085*	
H17B	0.8873	0.3637	0.5243	0.085*	
H17C	0.9152	0.2664	0.4103	0.085*	
O1	0.92692 (17)	0.81485 (16)	0.06163 (10)	0.0434 (4)	
O2	1.00158 (17)	0.74368 (16)	0.27742 (11)	0.0449 (4)	
O3	0.90251 (18)	0.49911 (17)	0.38942 (10)	0.0426 (4)	
O4	0.3099 (2)	−0.0118 (2)	0.39693 (13)	0.0637 (5)	
O5	0.51392 (17)	−0.05526 (15)	0.18353 (10)	0.0418 (4)	
S1	0.38731 (7)	0.39573 (6)	0.27505 (5)	0.0536 (3)	

### Atomic displacement parameters (Å^2^)

**Table:**

	*U*^11^	*U*^22^	*U*^33^	*U*^12^	*U*^13^	*U*^23^
C1	0.0339 (8)	0.0410 (9)	0.0268 (7)	−0.0138 (7)	0.0016 (6)	−0.0030 (6)
C2	0.0317 (8)	0.0386 (9)	0.0341 (8)	−0.0145 (7)	0.0038 (6)	−0.0020 (7)
C3	0.0356 (8)	0.0388 (9)	0.0329 (8)	−0.0154 (7)	−0.0012 (6)	−0.0075 (7)
C4	0.0352 (8)	0.0407 (9)	0.0268 (8)	−0.0111 (7)	0.0003 (6)	−0.0067 (6)
C5	0.0393 (9)	0.0349 (8)	0.0301 (8)	−0.0149 (7)	0.0010 (6)	−0.0021 (6)
C6	0.0315 (8)	0.0367 (8)	0.0289 (7)	−0.0119 (6)	0.0029 (6)	−0.0069 (6)
C7	0.0449 (9)	0.0405 (9)	0.0253 (7)	−0.0174 (7)	0.0006 (6)	−0.0057 (6)
C8	0.0430 (9)	0.0380 (9)	0.0314 (8)	−0.0172 (7)	0.0007 (6)	−0.0084 (6)
C9	0.0344 (8)	0.0391 (9)	0.0347 (8)	−0.0130 (7)	0.0016 (6)	−0.0094 (7)
C10	0.0538 (11)	0.0611 (13)	0.0480 (11)	−0.0191 (10)	0.0214 (9)	−0.0226 (9)
C11	0.0442 (10)	0.0529 (11)	0.0366 (9)	−0.0185 (8)	0.0090 (7)	−0.0094 (8)
C12	0.0341 (8)	0.0391 (9)	0.0300 (8)	−0.0121 (7)	0.0030 (6)	−0.0093 (6)
C13	0.0737 (14)	0.0382 (10)	0.0539 (12)	−0.0231 (10)	0.0185 (10)	−0.0036 (8)
C14	0.0922 (18)	0.0546 (13)	0.0543 (13)	−0.0253 (13)	0.0184 (12)	0.0081 (10)
C15	0.0553 (11)	0.0502 (11)	0.0403 (9)	−0.0253 (9)	−0.0041 (8)	0.0072 (8)
C16	0.0674 (14)	0.0547 (12)	0.0625 (13)	−0.0240 (11)	−0.0031 (10)	−0.0239 (10)
C17	0.0788 (15)	0.0702 (14)	0.0343 (9)	−0.0404 (12)	−0.0135 (9)	0.0104 (9)
O1	0.0514 (8)	0.0488 (8)	0.0388 (7)	−0.0296 (6)	−0.0044 (5)	0.0062 (6)
O2	0.0485 (8)	0.0448 (8)	0.0480 (7)	−0.0216 (6)	−0.0104 (6)	−0.0074 (6)
O3	0.0574 (8)	0.0486 (7)	0.0284 (6)	−0.0242 (6)	−0.0078 (5)	−0.0003 (5)
O4	0.0793 (11)	0.0662 (10)	0.0479 (8)	−0.0317 (9)	0.0283 (7)	−0.0076 (7)
O5	0.0534 (8)	0.0344 (7)	0.0382 (7)	−0.0164 (6)	0.0142 (5)	−0.0073 (5)
S1	0.0570 (4)	0.0406 (4)	0.0624 (4)	−0.0154 (3)	0.0180 (3)	−0.0209 (2)

### Geometric parameters (Å, º)

**Table:**

C1—C2	1.389 (2)	C11—C12	1.427 (2)
C1—C6	1.391 (2)	C12—O5	1.368 (2)
C1—H1	0.9300	C13—O5	1.432 (2)
C2—O1	1.3611 (19)	C13—C14	1.492 (3)
C2—C3	1.404 (2)	C13—H13A	0.9700
C3—O2	1.3778 (18)	C13—H13B	0.9700
C3—C4	1.392 (2)	C14—O4	1.444 (3)
C4—O3	1.3734 (19)	C14—H14A	0.9700
C4—C5	1.393 (2)	C14—H14B	0.9700
C5—C6	1.396 (2)	C15—O1	1.428 (2)
C5—H5	0.9300	C15—H15A	0.9600
C6—C7	1.471 (2)	C15—H15B	0.9600
C7—C8	1.335 (2)	C15—H15C	0.9600
C7—H7	0.9300	C16—O2	1.431 (3)
C8—C9	1.464 (2)	C16—H16A	0.9600
C8—H8	0.9300	C16—H16B	0.9600
C9—C12	1.359 (2)	C16—H16C	0.9600
C9—S1	1.7322 (17)	C17—O3	1.415 (2)
C10—C11	1.363 (3)	C17—H17A	0.9600
C10—S1	1.713 (2)	C17—H17B	0.9600
C10—H10	0.9300	C17—H17C	0.9600
C11—O4	1.360 (2)		
			
C2—C1—C6	120.60 (14)	O5—C13—C14	111.46 (17)
C2—C1—H1	119.7	O5—C13—H13A	109.3
C6—C1—H1	119.7	C14—C13—H13A	109.3
O1—C2—C1	124.50 (15)	O5—C13—H13B	109.3
O1—C2—C3	115.47 (14)	C14—C13—H13B	109.3
C1—C2—C3	120.02 (15)	H13A—C13—H13B	108.0
O2—C3—C4	120.04 (14)	O4—C14—C13	111.1 (2)
O2—C3—C2	120.78 (15)	O4—C14—H14A	109.4
C4—C3—C2	119.08 (14)	C13—C14—H14A	109.4
O3—C4—C3	115.81 (14)	O4—C14—H14B	109.4
O3—C4—C5	123.31 (15)	C13—C14—H14B	109.4
C3—C4—C5	120.88 (14)	H14A—C14—H14B	108.0
C4—C5—C6	119.71 (15)	O1—C15—H15A	109.5
C4—C5—H5	120.1	O1—C15—H15B	109.5
C6—C5—H5	120.1	H15A—C15—H15B	109.5
C1—C6—C5	119.70 (14)	O1—C15—H15C	109.5
C1—C6—C7	118.70 (14)	H15A—C15—H15C	109.5
C5—C6—C7	121.44 (14)	H15B—C15—H15C	109.5
C8—C7—C6	130.10 (14)	O2—C16—H16A	109.5
C8—C7—H7	115.0	O2—C16—H16B	109.5
C6—C7—H7	115.0	H16A—C16—H16B	109.5
C7—C8—C9	130.45 (14)	O2—C16—H16C	109.5
C7—C8—H8	114.8	H16A—C16—H16C	109.5
C9—C8—H8	114.8	H16B—C16—H16C	109.5
C12—C9—C8	125.05 (15)	O3—C17—H17A	109.5
C12—C9—S1	109.52 (12)	O3—C17—H17B	109.5
C8—C9—S1	124.81 (13)	H17A—C17—H17B	109.5
C11—C10—S1	111.34 (14)	O3—C17—H17C	109.5
C11—C10—H10	124.3	H17A—C17—H17C	109.5
S1—C10—H10	124.3	H17B—C17—H17C	109.5
O4—C11—C10	125.54 (17)	C2—O1—C15	117.07 (13)
O4—C11—C12	122.54 (17)	C3—O2—C16	114.95 (14)
C10—C11—C12	111.91 (18)	C4—O3—C17	117.00 (13)
C9—C12—O5	123.02 (14)	C11—O4—C14	111.51 (15)
C9—C12—C11	114.32 (16)	C12—O5—C13	112.21 (13)
O5—C12—C11	122.66 (16)	C10—S1—C9	92.85 (9)
			
C6—C1—C2—O1	179.31 (15)	S1—C9—C12—O5	177.21 (13)
C6—C1—C2—C3	0.5 (2)	C8—C9—C12—C11	−173.91 (17)
O1—C2—C3—O2	−3.6 (2)	S1—C9—C12—C11	−2.66 (19)
C1—C2—C3—O2	175.28 (15)	O4—C11—C12—C9	−178.64 (16)
O1—C2—C3—C4	−179.85 (14)	C10—C11—C12—C9	2.1 (2)
C1—C2—C3—C4	−1.0 (3)	O4—C11—C12—O5	1.5 (3)
O2—C3—C4—O3	4.7 (2)	C10—C11—C12—O5	−177.77 (17)
C2—C3—C4—O3	−179.03 (15)	O5—C13—C14—O4	63.0 (3)
O2—C3—C4—C5	−174.82 (15)	C1—C2—O1—C15	10.4 (3)
C2—C3—C4—C5	1.5 (3)	C3—C2—O1—C15	−170.77 (16)
O3—C4—C5—C6	179.03 (15)	C4—C3—O2—C16	−106.9 (2)
C3—C4—C5—C6	−1.5 (3)	C2—C3—O2—C16	76.9 (2)
C2—C1—C6—C5	−0.6 (2)	C3—C4—O3—C17	−179.64 (17)
C2—C1—C6—C7	−176.03 (15)	C5—C4—O3—C17	−0.2 (3)
C4—C5—C6—C1	1.0 (2)	C10—C11—O4—C14	−164.6 (2)
C4—C5—C6—C7	176.38 (15)	C12—C11—O4—C14	16.3 (3)
C1—C6—C7—C8	−155.46 (19)	C13—C14—O4—C11	−46.7 (3)
C5—C6—C7—C8	29.2 (3)	C9—C12—O5—C13	−166.87 (17)
C6—C7—C8—C9	9.0 (3)	C11—C12—O5—C13	13.0 (2)
C7—C8—C9—C12	−148.21 (19)	C14—C13—O5—C12	−43.6 (2)
C7—C8—C9—S1	41.8 (3)	C11—C10—S1—C9	−0.85 (18)
S1—C10—C11—O4	−179.73 (16)	C12—C9—S1—C10	2.00 (14)
S1—C10—C11—C12	−0.5 (2)	C8—C9—S1—C10	173.28 (16)
C8—C9—C12—O5	5.9 (3)		

### Hydrogen-bond geometry (Å, º)

**Table:**

*D*—H···*A*	*D*—H	H···*A*	*D*···*A*	*D*—H···*A*
C15—H15*B*···O1^i^	0.96	2.57	3.477 (3)	157
C17—H17*B*···O3^ii^	0.96	2.57	3.266 (3)	129

Symmetry codes: (i) −*x*+2, −*y*+2, −*z*; (ii) −*x*+2, −*y*+1, −*z*+1.
